# Targeting of multiple myeloma-related angiogenesis by miR-199a-5p mimics: *in vitro* and *in vivo* anti-tumor activity

**DOI:** 10.18632/oncotarget.1747

**Published:** 2014-03-14

**Authors:** Lavinia Raimondi, Nicola Amodio, Maria Teresa Di Martino, Emanuela Altomare, Marzia Leotta, Daniele Caracciolo, Annamaria Gullà, Antonino Neri, Simona Taverna, Patrizia D'Aquila, Riccardo Alessandro, Antonio Giordano, Pierosandro Tagliaferri, Pierfrancesco Tassone

**Affiliations:** ^1^ Department of Experimental and Clinical Medicine, Magna Graecia University and Medical Oncology Unit, T. Campanella Cancer Center, Salvatore Venuta University Campus, Catanzaro, Italy; ^2^ Department of Medical Sciences University of Milan, Hematology1, IRCCS Policlinico Foundation, Milan, Italy; ^3^ Department of Pathology and Forensic and Medical Biotechnology, Section of Biology and Genetics, University of Palermo, Italy; ^4^ Department of Biology, Ecology and Earth Science (DiBEST),University of Calabria, Arcavacata di Rende, Cosenza, Italy; ^5^ Sbarro Institute for Cancer Research and Molecular Medicine, Center for Biotechnology, College of Science and Technology, Temple University, Philadelphia, PA, USA

**Keywords:** miR-199-5p, microRNA, miRNA, multiple myeloma, plasma cell leukemia, microenviroment, hypoxia, angiogenesis

## Abstract

Multiple myeloma (MM) cells induce relevant angiogenic effects within the human bone marrow *milieu* (huBMM) by the aberrant expression of angiogenic factors. Hypoxia triggers angiogenic events within the huBMM and the transcription factor hypoxia-inducible factor-1α (HIF-1α) is over-expressed by MM cells. Since synthetic miR-199a-5p mimics negatively regulates HIF-1α, we here investigated a miRNA-based therapeutic strategy against hypoxic MM cells. We indeed found that enforced expression of miR-199a-5p led to down-modulated expression of HIF-1α as well as of other pro-angiogenic factors such as VEGF-A, IL-8, and FGFb in hypoxic MM cells *in vitro*. Moreover, miR-199a-5p negatively affected MM cells migration, while it increased the adhesion of MM cells to bone marrow stromal cells (BMSCs) in hypoxic conditions. Furthermore, transfection of MM cells with miR-199a-5p significantly impaired also endothelial cells migration and down-regulated the expression of endothelial adhesion molecules such as VCAM-1 and ICAM-1. Finally, we identified a hypoxia/AKT/miR-199a-5p loop as a potential molecular mechanism responsible of miR-199a-5p down-regulation in hypoxic MM cells. Taken together our results indicate that miR-199a-5p has an important role for the pathogenesis of MM and support the hypothesis that targeting angiogenesis via a miRNA/HIF-1α pathway may represent a novel potential therapeutical approach for this still lethal disease.

## INTRODUCTION

Multiple myeloma (MM) is characterized by abnormal proliferation of malignant plasma cells (PCs) within the bone marrow (BM), which leads to paraprotein release in the serum, osteolytic bone disease, anemia and renal failure [1]. Despite in the recent years important achievements in the understanding of MM pathogenesis have been obtained [2] and novel research platforms [3-5] and new therapeutics [6, 7] have been made available, MM invariably progresses to a lethal stage and is still an incurable malignancy. Disease progression is sustained by a supportive network which is based on complex cross-talks and involves a variety of cells of the human bone marrow *milieu* (huBMM), including endothelial, inflammatory and stromal cells. In this context, the BM angiogenesis plays a critical role in the MM pathogenesis and progression [8] and MM has been the first hematological disease in which the prognostic relevance of angiogenesis has been demonstrated [9, 10]. Interestingly, several studies indicate that monoclonal gammopathy of undetermined significance (MGUS) and non-active MM (smoldering MM) represent the avascular phase of PC malignancies, while active intramedullary MM is the vascular phase of the disease. In line with these data, increased BM micro-vessel density (BM-MVD) was observed in patients with active MM and was correlated to disease progression, adverse outcome and resistance to chemotherapy [11]. MM cells promote the angiogenic switch through the direct expression of angiogenic molecules or their induction in the BM stromal cells (BMSCs) within the huBMM [12]. In fact, BMSCs may cooperate with malignant PCs to produce pro-angiogenic factors, which finally induce full angiogenic events. In this contest, a critical role in the regulation of the angiogenic switch is played by the hypoxic huBMM: in fact, it is now becoming clear that MM cells are chronically exposed to low oxygen levels and abnormally activate hypoxia-inducible factors (HIFs) [11, 13]. The aberrant HIFs activation in turn increases the BM angiogenesis via up-regulation of VEGF-A, IL8 and CXCL12 [14]. In addition, the hypoxia supports MM cells survival, invasion, also contributing to disease progression and development of drug-resistance [15, 16].

Notably, HIF-1α suppression in myeloma cells blocks tumoral growth *in vivo* and interferes negatively with angiogenesis and bone destruction [17].

Recent findings have highlighted a relevant role for microRNAs (miRNAs) in the regulation of angiogenic events [18, 19]. miRNAs are short non-coding RNA molecules able to regulate gene expression, affecting the stability and/or translation of target mRNAs [20]. In MM, specific miRNA signatures have been associated to different steps of MM development from normal PCs via MGUS to clinically overt MM [21, 22]. Therefore a strong relationship between deregulated expression of miRNAs and the tumor phenotype has been demonstrated [21-26] and miRNA deregulation has been associated to the typical chromosomal aberrations [21, 22, 27, 28]. More recently, miRNAs beyond their key role in MM pathogenesis, are emerging as potential tools for the targeting the miRNA network as a novel therapeutic strategy providing a novel rationale and a new venue of investigation in this disease [29-37]. There is now strong evidence that hypoxia controls miRNAs expression in cancer [38-40]; in turn, hypoxia-regulated miRNAs interfere with a large variety of processes such as angiogenesis, apoptosis, proliferation and migration [39]. Among miRNAs deregulated in MM, miR-199a-5p is of relevant interest because directly targets HIF1-α, a prominent transcription factor which regulates angiogenesis, predominantly via induction of VEGF transcription [41-43]. Furthermore, it has been demonstrated that hypoxia induces down-regulation of miR-199a-5p, probably through activation of the AKT pathway [44, 45].

On these premises, we investigated the functional role of miR-199-5p in MM. We first evaluated its expression in a panel of MM cell lines and then we studied the biological effect induced *in vitro* by enforced expression of synthetic miR-199a-5p mimics in both normoxic and hypoxic conditions. Moreover, we studied the anti-tumor potential of *in vivo* delivered of miR-199a-5p against human MM xenografts in mice. We believe that our results disclose a relevant role of miR-199a-5p in MM-angiogenesis and provide the rationale for the design of innovative miRNA-based therapeutic approach in this disease.

## RESULTS

### miR-199a-5p expression in human MM cell lines and hypoxic-response of MM cells

We first evaluated the miR-199a expression profile in a set of MM cell lines (OPM2, U266, KMS11, MM1S, RPMI 8266, KMS34, INA6, KMS-12M, NCI-H929, SKMM1) as compared to normal BM-derived CD138^+^ PCs from healthy donors.

**Figure 1:**
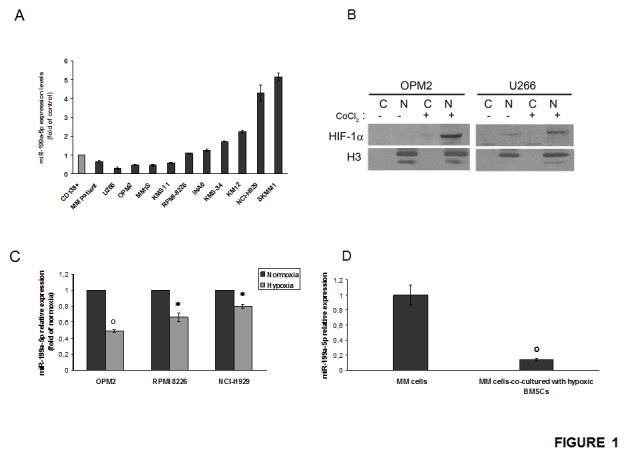
miR-199a-5p expression in myeloma cells and hypoxic-effect on miR-199a-5p expression in MM cells (a) Quantitative RT-PCR analysis of miR-199a-5p using total RNA from 10 MM cell lines and 1 MM patient sample. Raw Ct values were normalized to RNU44 housekeeping snoRNA and expressed as fold increase over control CD138^+^ cells (black column, 1 arbitrary unit). Columns, means; Bars, S.D Values represent mean of three different experiments. (b) Western blotting analysis showing HIF-1α expression in nuclear (N) and cytoplasmic (C) -enriched cell fractions of MM cell lines treated for 4 hours with 100µM/L of the hypoxia-mimicking Cobalte Chloride. Histone H3 was used as loading control to discriminate the different cell fractions. (c) Quantitative RT-PCR of miR-199a-5p in OPM2, NCI-H929 and RPMI-8226 MM cell lines cultured in both normoxic and hypoxic conditions. Raw Ct were normalized to RNU44 housekeeping snoRNA and expressed as fold increase over normoxic MM cells (black column, 1 arbitrary unit). Columns, means; Bars, S.D. Values represent mean of three different experiments. P values were obtained using two-tailed *t* test. *P<0,05; °P<0,01 (d) Quantitative RT–PCR of miR-199a-5p in OPM2 cells co-cultured for 24 hours with BMSCs and then immunopurified by immunomagnetic sorting with anti-CD138 beads. Raw Ct values were normalized to RNU44 housekeeping snoRNA and expressed as fold increase over normoxic OPM2 cells (black column, 1 arbitrary unit). Columns, means; Bars, S.D. Values represent mean of three different experiments. °P<0,01.

Among cell lines analyzed by qRT-PCR, we found that miR-199a-5p is significantly down-regulated in 4 (OPM2, U266, KMS11, MM1S) out of 10 lines as compared to normal PCs (Fig. 1A).

To study the response of MM cells to hypoxia, we reproduced an hypoxic condition by the use of cobalt chloride or by culturing cells for at least 24 hours in a 1% O_2_ atmosphere. By western blotting analysis of nuclear-enriched extracts from CoCl_2_-stimulated MM cells, we found that hypoxic conditions triggered the increase of HIF-1α levels in MM cells (Fig.1B). Similar results were obtained when MM cells were cultured in a hypoxia chamber (data not shown).

In parallel, we analyzed the expression of miR-199a-5p in response to hypoxia in MM cells. To this end, we used MM cell lines with different basal levels of miR-199a-5p in standard conditions. We observed a significant decrease of miR-199a-5p expression in hypoxic MM cells as compared to normoxic counterpart (Fig. 1C). Notably, miR-199a-5p expression was further decreased when MM cells where co-cultured with hypoxic BMSCs (Fig. 1D). These results indicate that hypoxia in MM cells down-regulate miR-199a-5p and this effect is further strengthened by an hypoxic huBMM.

### miR-199a-5p targets and suppresses HIF-1α expression into hypoxic MM cells

**Figure 2:**
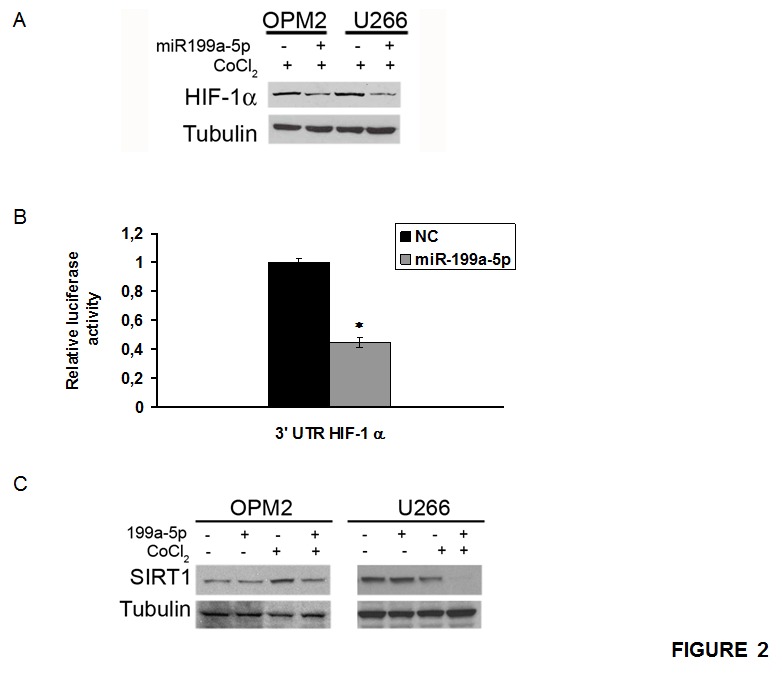
miR-199a-5p targets HIF-1α and reduces HIF-1α and SIRT1 protein expression in hypoxic MM cells (a) Western blotting analysis of HIF-1α 24 hours after transfection with synthetic miR-199a-5p (miR-199a-5p) or scrambled oligonucleotides (NC) in U266 and OPM2 cell lines treated for 4 hours with 100µM/L of the hypoxia-mimicking Cobalte Chloride. Tubulin was used as loading control. (b) Dual luciferase assay of OPM2 cells co-transfected with firefly luciferase constructs containing the 3'UTR of HIF-1α and miR-199a-5p or scrambled oligonucleotides (NC) as indicated. The firefly luciferase activity was normalized to renilla luciferase activity. The data are shown as relative luciferase activity of miR-199a-5p-transfected cells as compared to the control (NC) of a total of six experiments from three independent transfections. P<0,05. (c) Western blotting analysis of SIRT1 24 hours after transfection with synthetic miR-199a-5p (miR-199a-5p) or scrambled oligonucleotides (NC) in U266 and OPM2 cell lines treated for 4 hours with 100µM/L of the hypoxia-mimicking Cobalte Chloride. Tubulin was used as loading control.

To assess whether miR-199a-5p directly affects the expression of HIF-1-α in MM cells, we evaluated the effect induced by enforced expression of synthetic miR-199a-5p mimics on HIF-1-α protein levels in cells exposed to hypoxic culture conditions. By qRT–PCR, we first measured the transfection efficiency of miR-199a-5p 24h after treatment (Supplemental Figure 1A). Notably, we observed that enforced expression of miR199a-5p into hypoxic MM cells strongly suppressed nuclear HIF-1α protein expression (Fig. 2A).

To validate this interaction in MM cells, OPM2 were co-transfected with synthetic miR- 199a-5p or scrambled oligonucleotides (NC), together with an expression vector carrying the 3'UTR of HIF-1α mRNA cloned downstream to the luciferase reporter gene. Figure 2B clearly shows a significantly lower luciferase activity in OPM2 cells transfected with miR-199a-5p mimics as compared to the control.

Moreover, we investigated the effect induced on the mammalian class-III protein deacetylase of the sirtuin family (Sirt1), a putative target of miR-199a-5p which plays an important role in stabilizing HIF-1α. As shown in Fig. 2C, miR-199a-5p indeed reduced Sirt1 protein expression.

### miR-199a-5p inhibits the hypoxic induction of pro-angiogenic factors and reduces endothelial cells migration

**Figure 3:**
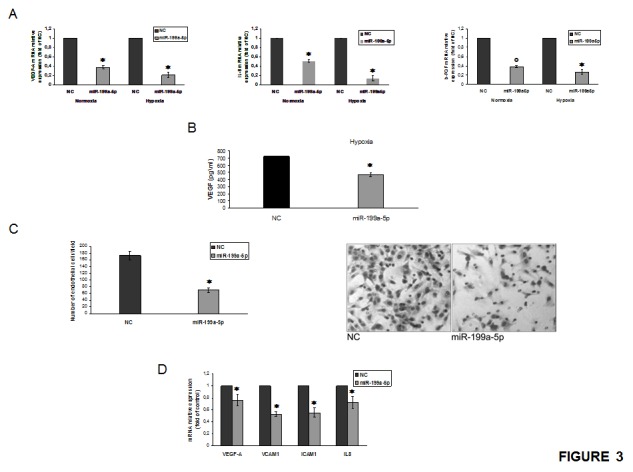
miR-199a-5p inhibits the hypoxia-induction of pro-angiogenic factors and reduces endothelial cells migration (a) Quantitative RT-PCR of VEGF-A, IL-8 and b-FGF in both normoxic and hypoxic OPM2 cells transfected with synthetic miR-199a-5p (miR-199a-5p) or scrambled oligonucleotides (NC). Raw Ct were normalized to b-actin housekeeping and expressed as fold increase of negative control (black column, 1 arbitrary unit). Columns, means; Bars, S.D. Values represent mean of three different experiments. °P<0,01; *P<0,05. (b) Vascular endothelial growth factor (VEGF-A) protein production in the conditioned medium derived from hypoxic OPM2 cells transfected with synthetic miR-199a-5p (miR-199a-5p) or scrambled oligonucleotides (NC) was determined by enzyme-linked immunoadsorbent assay (ELISA). Data presented are the mean of three separate experiments. *P<0,05. (c) Chemotaxis assay of endothelial cells (HUVEC) exposed to conditioned medium from OPM2 cells transfected with either scrambled (miR-NC) or synthetic pre-miR-199a-5p (miR-199a-5p) (right panel); Huvec cells treated as in (c) and observed at contrast phase microscopy (left panel). *P<0,05. (d) Quantitative RT-PCR of VEGF-A, VCAM1, ICAM1 and IL-8 in Huvec cells exposed for 24 hours to conditioned medium from OPM2 cells transfected with synthetic miR-199a-5p (miR-199a-5p) or scrambled oligonucleotides (NC). Raw Ct were normalized to b-actin housekeeping and expressed as fold increase of negative control (black column, 1 arbitrary unit). Columns, means; Bars, S.D. Values represent mean of three different experiments. *P<0,05.

qRT-PCR basal analysis of hypoxic MM cells revealed that VEGF-A and IL-8 mRNA expression were about 8 and 10-fold higher as compared to normoxic cells (Supplemental Fig. 2A). Moreover, we found that mRNA basic-fibroblast growth factor-2 (bFGF) expression was approximately 5-fold higher in hypoxic as compared to normoxic cells (Supplemental Fig. 2A). On the basis of these findings, we attempted to restore the miR-199a-5p expression in MM cells in order to evaluate its activity on the expression of pro-angiogenic factors. We indeed found that enforced expression of miR199a-5p mimics significantly reduced VEGF-A, IL-8 and bFGF mRNA, both in normoxic as well as in hypoxic cells (Fig. 3A). Taking in account that secretion is increased in hypoxic MM cells, we investigated whether enforced expression of miR-199a-5p in hypoxic MM cells was able to reduce VEGF-A protein secretion. Figure 3B shows that miR-199a-5p transfection in myeloma cells significantly decreased VEGF-a protein secretion

To investigate the role of miR-199a-5p in endothelial cells migration, we performed a chemotaxis assay. We observed that miR-199a-5p mimics produced a relevant decrease in the number of endothelial migrating cells in normoxic condition (Fig. 3C). Furthermore, addition of normoxic conditioned medium from MM cells over-expressing miR-199a-5p to the endothelial monolayer reduced the expression of cell-cell adhesion molecules such as VCAM-1 and ICAM-1 (Fig. 3D). Accordingly, the expression of VEGF-A and IL-8 mRNA produced by endothelial cells, were also significantly decreased (Fig.3D).

Taken together, these results indicate that miR-199a-5p directly inhibits the synthesis of angiogenic factors by MM cells and interferes with the MM/endothelial cell loop which promotes the basic events in the angiogenic response.

### miR-199a-5p regulates DDR1 expression and decreases chemotaxis of MM cells

**Figure 4:**
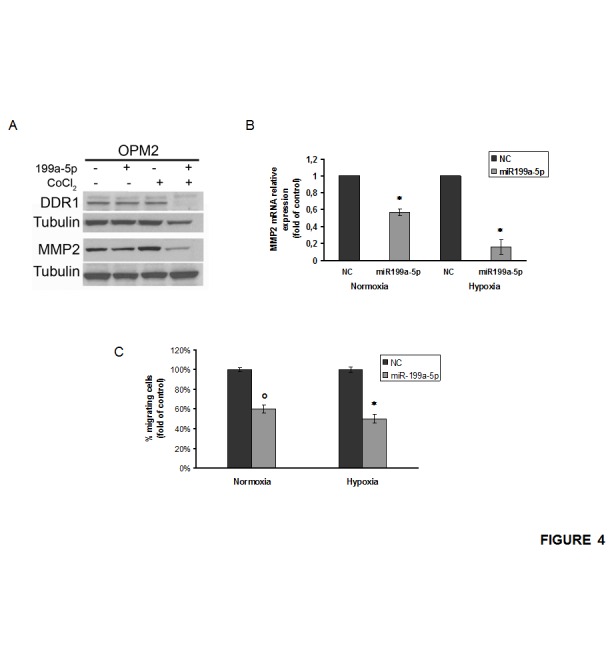
miR-199a-5p regulates DDR1 expression and decreases migration of MM cells (a) Western blotting analysis of DDR1 and MMP2 24 hours after transfection with synthetic miR-199a-5p (miR-199a-5p) or scrambled oligonucleotides (NC) in OPM2 cells treated for 4 hours with 100µM/L of the hypoxia-mimicking Cobalte Chloride. Tubulin was used as loading control. (b) Quantitative RT-PCR of matrix metalloproteinase-2 (MMP2) in both normoxic and hypoxic OPM2 cells transfected with synthetic miR-199a-5p (miR-199a-5p) or scrambled oligonucleotides (NC). Raw Ct were normalized to b-actin housekeeping and expressed as fold increase of negative control (black column, 1 arbitrary unit). Columns, means; Bars, S.D. Values represent mean of three different experiments. (c) Chemotaxis assay of both normoxic and hypoxic OPM2 cells transfected with synthetic miR-199a-5p (miR-199a-5p) or scrambled oligonucleotides (NC). °P0,01.; *P<0,05.

The discoidin domain receptor-1 (DDR1) is a predicted target of miR-199a-5p [41]. Here we investigated if its expression was regulated by miR-199a-5p in MM cells. We analyzed DDR1 expression in both normoxic and hypoxic MM cells transfected with miR-199a-5p. Western blot analysis demonstrated a strong reduction of DDR1 expression in hypoxic MM cells (Fig. 4A). qRT-PCR basal analysis of hypoxic MM cells revealed that MMP2 mRNA expression was about 5-fold higher as compared to normoxic cells (Supplemental Fig. 2B). Conversely, we found a decrease in metalloproteinase MMP2 also, at mRNA and protein level, both in normoxic and hypoxic MM cells transfected with miR-199a-5p, although these latter effects were mainly detectable in hypoxic conditions (Fig. 4A-B).

To assess the effect of miR-199a-5p expression on the migration of MM cells, we performed a chemotaxis assay on hypoxic and normoxic MM cells. Figure 4C shows that miR-199a-5p transfection reduces the percentage of MM migrating cells both in normoxic and in hypoxic conditions.

### miR-199a-5p induces adhesion to hypoxic BMSCs

**Figure 5:**
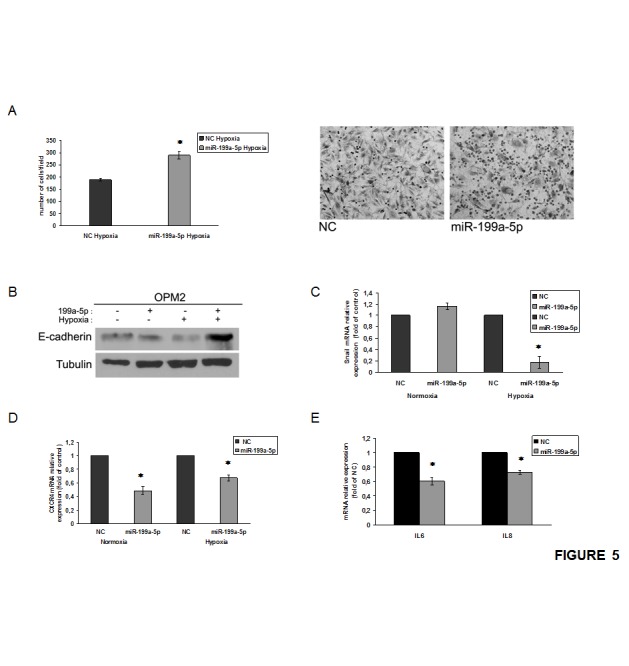
miR-199a-5p overexpression increases adhesion of MM cells to a monolayer of BMSCs under hypoxic conditions (a) Adhesion assay of hypoxic OPM2 cells transfected with synthetic miR-199a-5p (miR-199a-5p) or scrambled oligonucleotides (NC) and seeded on to hypoxic BMSCs monolayer (right panel); OPM2 cells treated as in (a) and observed at contrast phase microscopy (left panel) *P<0,05. (b) Western blotting analysis of E-cadherin 24 hours after transfection with synthetic miR-199a-5p (miR-199a-5p) or scrambled oligonucleotides (NC) in OPM2 cells. Tubulin was used as loading control. (c) Quantitative RT-PCR of Snail in both normoxic and hypoxic OPM2 cells transfected with synthetic miR-199a-5p (miR-199a-5p) or scrambled oligonucleotides (NC). Raw Ct were normalized to b-actin housekeeping and expressed as fold increase of negative control (black column, 1 arbitrary unit). Columns, means; Bars, S.D. Values represent mean of three different experiments. *P<0,05. (d) Quantitative RT-PCR of CXCR4 in both normoxic and hypoxic OPM2 cells transfected with synthetic miR-199a-5p (miR-199a-5p) or scrambled oligonucleotides (NC). Raw Ct were normalized to b-actin housekeeping and expressed as fold increase of negative control (black column, 1 arbitrary unit). Columns, means; Bars, S.D. Values represent mean of three different experiments. *P<0,05. (e) Quantitative RT-PCR of IL6 and IL8 in normoxic HS5 cells exposed for 24 hours to conditioned medium from hypoxic OPM2 cells transfected with synthetic miR-199a-5p (miR-199a-5p) or scrambled oligonucleotides (NC). Raw Ct were normalized to b-actin housekeeping and expressed as fold increase of negative control (black column, 1 arbitrary unit). Columns, means; Bars, S.D. Values represent mean of three different experiments. *P<0,05.

In order to investigate the role of miR-199a-5p in the adhesion of MM cells to BMSCs, we first found that hypoxia reduces the adhesion of MM cells to a BMSCs monolayer (Supplemental Fig. 3A). Conversely, we showed that the enforced expression of miR-199a-5p reconstituted the adhesion capacity of hypoxic MM cells to a monolayer of hypoxic BMSCs (Fig.5A). Moreover, hypoxic MM cells transfected with miR-199a-5p expressed higher levels of E-cadherin and lower levels of Snail mRNA (Fig.5B-C). Supplemental figure 3B shows that the expression of CXCR4 mRNA was increased in hypoxic MM cells, while mR-199a-5p transfection decreased CXCR4 mRNA levels (Fig. 5D). In addition, BMSCs exposed to conditioned medium of miR-199a-5p-transfected MM cells showed impaired expression of IL6 and IL8. Taken together these findings demonstrate that miR199a-5p is able to reverse the invasive phenotype induced by hypoxic BM microenvironment.

### miR-199a-5p decreases cell proliferation and increases apoptosis in hypoxic conditions

**Figure 6:**
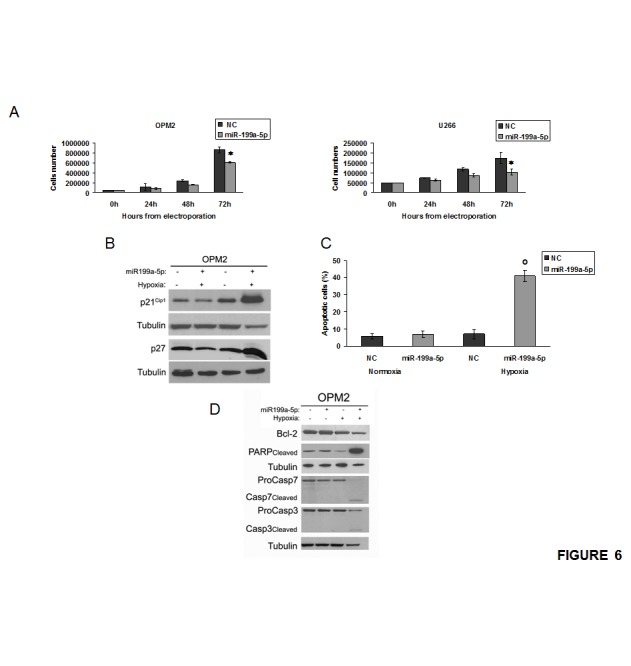
miR-199a-5p overexpression reduces proliferation and promotes apoptosis in hypoxic MM cells (a) Cell growth curves of OPM2 and U266 transfected with synthetic miR-199a-5p (miR-199a-5p) or scrambled oligonucleotides (NC). Averaged values of three independent experiments are plotted including ±S.D. *P<0,05. (b) Western blotting analysis of p21^Cip-1^ and p27^Kip-1^ 24 hours after transfection with synthetic miR-199a-5p (miR-199a-5p) or scrambled oligonucleotides (NC) in both normoxic and hypoxia-induced OPM2 cells. Tubulin was used as loading control. (c) Annexin V-staining of OPM2 cells 48 hours after transfection with synthetic miR-199a-5p or scrambled oligonucleotides (NC). The percentage of Annexin-V-positive cells is reported. Data are the average of three independent experiments ±S.D. °P<0,01. (d) Western blotting analysis of proteins caspase 3/7, cleaved poly ADP-ribose polymerase (PARP) and Bcl2, 24 hours after transfection with synthetic miR-199a-5p (miR-199a-5p) or scrambled oligonucleotides (NC) in both normoxic and hypoxia-induced OPM2 cells. Tubulin was used as loading control.

To evaluate the effects induced by enforced expression of miR-199a-5p on MM cell growth, we analyzed cell proliferation, cell cycle and apoptosis. As shown in Figure 6A, enforced expression of miR199a-5p decreased cell growth in all MM cell lines analyzed; in particular, cell growth inhibition was more evident 72 hours after transfection. Cell-cycle analysis showed that miR-199a-5p induced only a slight increase in G1 and a decrease in S-G2 phases under hypoxic conditions (Data not shown). Notably, we found up-regulation of the cell-cycle inhibitors p21^Cip-1^ and p27^kip-1^, even if this effect occurred only in hypoxic MM cells (Fig. 6B). Furthermore, flow cytometry demonstrated that miR-199a-5p induced apoptosis only in hypoxic MM cells (Fig. 6C). Western blot analysis revealed that miR-199a-5p-induced apoptosis was associated to activation of pro-apoptotic proteins caspase 3/7 and cleaved poly ADP-ribose polymerase (PARP), as well as reduction of the anti-apoptotic BCL-2 protein (Fig. 6D). All together these findings provide evidence that enforced expression of miR-199a-5p exert antitumor activity in conditions that recapitulate the MM hypoxic huBMM.

### AKT pathway regulates miR-199a-5p activity in hypoxia

**Figure 7:**
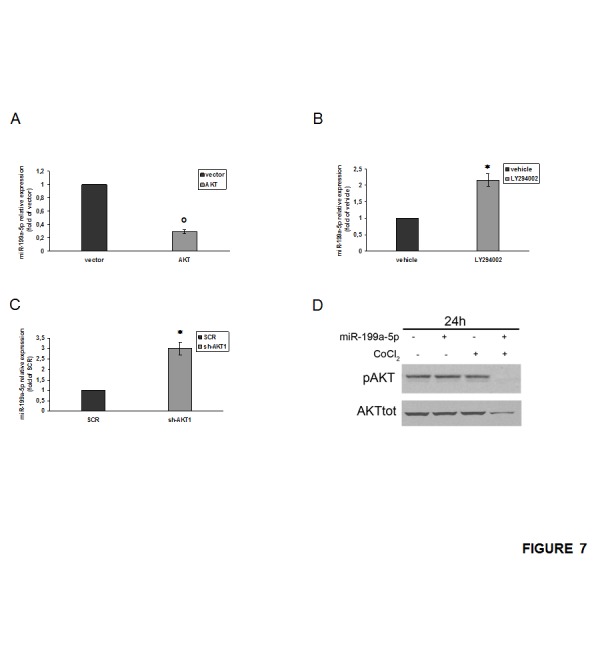
AKT control of miR-199a-5p expression in hypoxic MM cells (a) NCI-H929 cells were electroporated with the pcDNA.3.1-HA-myr-AKT dominant active construct (AKT) or the empty vector pcDNA3.1 (vector) and 48 hours later analyzed for miR-199a-5p expression levels by quantitative RT–PCR. Raw Ct values were normalized to RNU44 housekeeping snoRNA and expressed as fold increase over vector (black column, 1 arbitrary unit). Columns, means; Bars, S.D Values represent mean of three different experiments. °P<0,01. (b) Quantitative RT–PCR of miR-199a-5p in NCI-H929 cells treated with 20 µM LY294002 or vehicle (DMSO) for 48 hours. Raw Ct values were normalized to RNU44 housekeeping snoRNA and expressed as fold increase over vehicle (black column, 1 arbitrary unit). Columns, means; Bars, S.D Values represent mean of three different experiments. P<0,05. (c) NCI-H929 cells were electroporated with an AKT short hairpin (sh)RNA- or control (SCR) shRNA plasmids and 48 hours later analyzed for miR-199a-5p expression levels by quantitative RT–PCR. Raw Ct values were normalized to RNU44 housekeeping snoRNA and expressed as fold increase over control (SCR) (black column, 1 arbitrary unit). Columns, means; Bars, S.D Values represent mean of three different experiments. P<0,05. (d) Western blotting analysis of total and phospho-AKT 24 hours after transfection with synthetic miR-199a-5p (miR-199a-5p) or scrambled oligonucleotides (NC) in OPM2 cells treated for 4 hours with 100µM/L of the hypoxia-mimicking Cobalte Chloride. Tubulin was used as loading control.

To investigate the molecular mechanism of miR-199a-5p down-regulation in hypoxic MM cells, we over-expressed AKT1 in MM cells and we evaluated for the miR-199a-5p expression. As shown in Fig. 7A, over-expression of AKT1 induced down-regulation of miR-199a-5p. Furthermore, when MM cells were treated with the PI3-K/AKT inhibitor LY294002, we observed restoration of miR-199a-5p expression (Fig.7B). In addition, we silenced transiently AKT1 in MM cells and we found an increase of miR-199a-5p expression (Fig. 7C). Interestingly, after miR-199a-5p transfection in MM cells, we found a reduction of total and phosphorylated AKT1 protein levels (Fig. 7D). These data indicate the occurrence of a negative feedback loop between miR-199a-5p and AKT pathway where miR-199a-5p negatively regulates AKT expression, while activation and functional AKT represses miR-199a-5p expression (Supplemental Fig. 4A).

Another mechanism that control the expression of miR-199a is the methylation status of its promoters on both Chr1 and Chr19 [42, 46]. To investigate possible epigenetic mechanisms involved in the down-modulated expression of miR-199a-5p, we treated MM cells with the DNA demethylating agent 5-aza-dC [47]. This experiment was performed in three different cell lines expressing different levels of miR199a-5p and we found that miR-199a-5p expression was increased only in the two cell lines where its levels are low (Supplemental Figure 5A). In contrast, no differences were observed in cells where miR199a-5p is not down-regulated.

In order to clarify if down-regulation of miR-199a-5p in MM cell lines specifically depend by hypermethylation of its two promoter regions in chromosome 19 (1a) and in chromosome 1 (2a), respectively, the methylation levels of CpG sites located within these regions were evaluated by Sequenom MassARRAY EpiTYPER. To this aim, OPM2, U266 and NCI-H929 MM cells were treated with 1μM 5-azacytidine or with vehicle (RPMI medium) [36].

We observed that the methylation status of the promoter regions remained basically unchanged, (Supplemental Fig. 5B) despite that the azacytidine treatment increased miR-199a-5p levels in OPM2 and U266 MM cells (data not shown).

In order to further verify if the demethylation of miR-199a promoters was dose-dependent, we treated U266 cell line with either 5μM 5-azacytidine or with trichostatin A, a histone deacetylase inhibitor with demethylating activity. Again we did not observe demethylation of miR-199a-5p (Supplemental Fig. 5C) of both promoter regions.

These results suggest that the increase of miR-199a-5p expression following treatment with 5-AZA does not involve the de-methylation of mir199a-5p promoters thus suggesting an alternative as yet unidentified epigenetic mechanism.

### *In vivo* anti-tumor activity of formulated miR-199a-5p against MM xenografts

**Figure 8:**
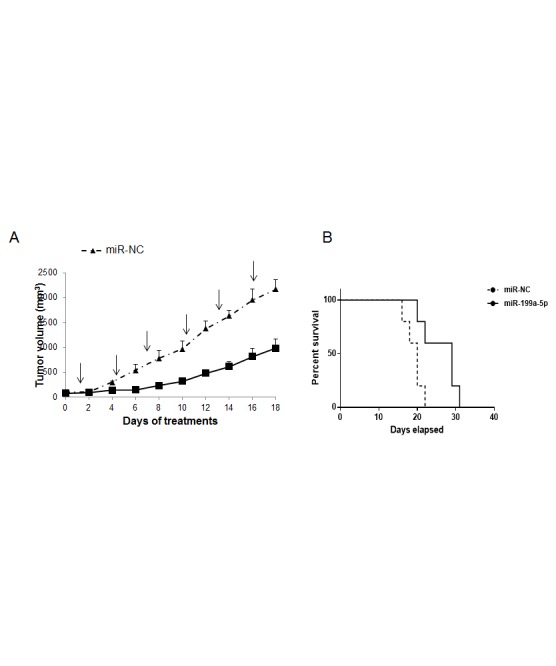
*In vivo* anti-tumor activity of miR-199a-5p after intratumoral delivery in MM mouse-models (a) *In vivo* tumor growth of NCI-H929 xenografts intratumorally-treated with NLE (MaxSuppressor™ In Vivo RNA-Lancer II)-miR-199a-5p or miR-NC. Palpable subcutaneous tumor xenografts were treated every 3 days (indicated by arrows) for a total of six injections, with 20 µg of formulated miR-199a-5p or miR-NC. Tumors were measured with an electronic caliper every two days, averaged tumor volume of each group ±S.D. are shown. P<0,05. (b) Survival curves (Kaplan-Meier) of intratumorally miR-199a-5p treated mices show prolongation of survival compared to miR-NC (log-rank test, P=0.023). Survival was evaluated from the first day of treatment until death or sacrifice. Percent of mice alive is shown.

We next investigated the effect of miR-199a-5p treatment on MM xenograft growth in NOD-SCID mice. miR-199a-5p or miR-NC were administered with NLE particles, a formulation specifically designed for the delivery of oligonucleotides *in vivo*. A highly significant (P<0,05) inhibition of tumor growth was detected following 6 injections (3 days apart) of miR-199a-5p formulated in NLE particles in NCI-H929 xenografts (Fig. 8A). Furthermore, we observed a prolongation of survival (*P=0.02*) of mice treated with miR-199a-5p mimics compared to control groups (median survival was 29 days *versus* 20 days in the control group) (Fig. 8B). We therefore conclude that miR-199a-5p by intratumoral delivery is highly effective against MM xenografts and significantly prolongs host survival.

## DISCUSSION

MM growth and progression is characterized by relevant angiogenic events that occur within the huBBM chronically exposed to low oxygen levels [11, 48, 49]. The BM hypoxia triggers the expression of a variety of genes essential for adaptation, cell invasion, dissemination and drug-resistance of MM cells [50]. Indeed, the hypoxia inducible-factor 1-α (HIF-1α), the master regulator of cellular response to hypoxia, is known to be abnormally activated in the BM of MM patients. Aberrant HIF1-α activation in MM cells contributes to the pathogenesis of the disease increasing angiogenesis via up-regulation of IL-8 and VEGF-A [8]. In this scenario, a variety of miRNAs have been found to play a relevant role as regulators of huBMM angiogenic events [51-53].

Here, we focused on the role of miR-199a-5p in both normoxic and hypoxic MM cells, because HIF-1α is predicted to be a potential target of miR-199a-5p [54]. It has been reported that miR-199a-5p is down-regulated in hypoxic conditions and specifically targets the 3'-UTR region of HIF-1α [55]. Importantly, miR-199a-5p is down-regulated in a variety of malignancies including MM where its deregulation seems to be correlated with chromosomal aberrations [54, 56]. Consistent with these observations, we found a deregulated expression of miR-199a-5p in myeloma cell lines.

To investigate the potential role of miR-199a-5p in a pathological hypoxic context, we selected MM cell lines with lower levels of miR-199a-5p as compared to normal CD138^+^ cells. Using these cells, we found a reduction of miR-199a-5p expression in both cells exposed to hypoxic conditions and co-cultured with hypoxic BMSCs. Importantly, we showed that enforced expression of miR-199a-5p significantly down-regulated the expression of HIF-1α in hypoxia-induced MM cells. In addition, we validated HIF-1α as a direct target of miR-199a-5p in myeloma cells. Accordingly, we found a strong reduction of Sirt1 expression, another miR-199a-5p predicted target required for HIF-1α accumulation [55, 57]. Interestingly, among the pro-angiogenic genes directly regulated by HIF-1α in MM cells, VEGF-A and IL-8 were strongly reduced after miR-199a-5p transfection, mainly in hypoxic conditions [11, 58, 59]. Consistent with these results, conditioned medium by miR-199a-5p-transfected hypoxic cells impaired IL-6 secretion by BMSCs. Consistent with the previously reported findings, the over-expression of miR-199a-5p in MM cells caused a decrease of bFGF expression, an important pro-angiogenic factor involved also in endothelial cell migration [60-63]. In fact, miR-199a-5p transfected MM cells reduced the migration of endothelial cells. Notably, this effect was associated to a significantly decreased expression of cell-cell adhesion molecules in endothelial cells such as VCAM-1 and ICAM-1. All together, these results suggest that restoration of miR-199a-5p has an important effect on myeloma-induced angiogenesis, acting on the production of pro-angiogenic factors by cells of the hypoxic microenvironment.

The discoidin domain receptor-1 (DDR1) is a collagen tyrosine kinase over-expressed in several human cancers [64]. Several studies suggested a potential role of DDR1 in tumor cell migration and invasion, usually mediated by extracellular matrix degradation; in addition, also DDR1 mRNA is predicted to be a potential target of miR-199a-5p [41]. In our study we found that repression of DDR1 protein upon enforced expression of miR-199a-5p occurred together with a reduced cellular migration and reduced expression of the matrix metalloproteinase MMP2. In parallel, miR-199a-5p reduces the expression of CXCR4 in hypoxic conditions.

A recent report demonstrated that hypoxia reduces adhesion of MM cells to the BM stroma thus promoting dissemination [65]. In detail, the authors showed clearly that the mechanism by which hypoxia leads to de-adhesion of MM cells to the BM stroma is mediated by E-cadherin, one of the most important molecules in cell-cell adhesion [65]. In an hypoxic context, HIF-1α recognizes Snail gene promoter leading to a decrease of E-cadherin expression thus resulting in an impairment of cell-cell adhesion and promotion of motility, invasion and metastasis [66-68].

In our study, we first validated HIF-1α as direct target of miR-199a-5p and then we evaluated if miR-199a-5p restoration in MM cells could have a role in the mechanism described above.

Hypoxic MM cells were transfected with miR-199a-5p and, once HIF-1α was blocked, we observed the inhibition of Snail expression and higher levels of E-cadherin.

We provide the first evidence, at our knowledge, that enforced expression of miR-199a-5p increases the percentage of hypoxic MM cells adherent to a monolayer of hypoxic BMSCs.

Taken together, these data suggest that *i*) miR199a-5p has a role in regulating tumor dissemination induced by hypoxia, and that *ii*) miR-199a-5p modulates tumor cell migration, at least in part by targeting DDR1.

Furthermore, we demonstrated that the enforced expression of miR-199a-5p triggers anti-proliferative and pro-apoptotic effects in hypoxic MM cells. Importantly, all our observations translated in a significant *in vivo* activity of formulated synthetic miR-199a-5b delivery which add potential clinical significance to our study. Together with the significant inhibition of tumor growth, we indeed achieve a clear advantage in terms of mice survival. This result is of great interest since conventional MM xenografts are based on aggressive established cell lines which produce quickly growing tumors poorly vascularized providing therefore an *in vivo* suitable informative system predictive of therapeutic response.

On the basis of this finding demonstrating a role for miR-199a-5p in MM angiogenic response and control of cell growth and survival, we investigated the molecular mechanism responsible of the miR-199a-5p downregulation in MM. It is well known that exposure to hypoxia elicits an adaptive response that also involves activation of the AKT pathway. It is well known that AKT enhances survival and anti-apoptotic gene expression acting also through miRNA-dependent mechanisms [69]. In particular, AKT induces downregulation of miR-199a-5p, which is required for de-repression of Hif-1α [45, 55]. Here, we demonstrated that functional AKT1 overexpression is sufficient in repressing miR-199a-5p expression while AKT1 depletion results in an increase of miR-199a-5p. Notably, miR-199a-5p induced also a reduction of total and phosphorylated AKT proteins expression. Although the precise molecular mechanisms underlying miR-199a-5p/Akt in MM needs to be fully elucidated, these results provide insights on miR-199a-5p-Akt pathway cross-talk in MM indicating the occurrence of a regulatory negative loop where miR-199a-5p downregulates functional AKT which, in turn, represses miR-199-5p [70]. Recent findings indicate that miR-199a expression is finely regulated also by promoter methylation on both Chr1 and Chr19, where its gene is located. Indeed, studies in several cancer cell lines showed that both promoter regions on Chr1 and Chr19 were hypermethylated [46, 71, 72]. Our findings indicate that treatment with demethylating agent can induce a restoration of miR-199a-5p expression in MM cells even if we can rule out, at least in our experimental conditions, a direct correlation with the methylation status of its promoter.

Treatments targeting the MM hypoxic niche are currently considered novel and promising therapeutics strategies.

In summary, our results revealed that hypoxia down-regulates the expression of miR-199a-5p in MM via activation of AKT in order to allow the up regulation of HIF-1α and pro-angiogenic genes. Our investigation provides evidence on miR-199a-5p involvement in cell proliferation, migration and apoptosis and suggests a critical role for miR-199a-5p in the hypoxic tumor microenvironment. Taken together, these data support the development of miR-199a-5p mimics as a new potential therapeutic agents in the treatment of MM. The important interaction with AKT pathway opens new opportunities for combinatory therapeutical approaches which may result in a selective and highly efficient targeting of pathways crucially involved in the control of MM cell growth and survival in the huBMM.

## MATERIALS AND METHODS

### Cell Lines, Primary Cells and Drugs

MM cell lines were purchased from ATTC and grown in RPMI-1640 (Gibco, Life Technologies, Carlsbad, CA, USA) supplemented with 10% fetal bovine serum (Lonza Group, Basel, Switzerland). HUVEC were obtained from Lonza (Clonetics, Verviers, Belgium) and grown in endothelial growth medium (EGM) according to supplier's information.

Stromal HS-5 cells were purchased from ATCC and cultured in Dulbecco's modified Eagle's medium, supplemented with 10% fetal bovine serum.

To induce hypoxia conditions, cells were placed in a modular incubator chamber (STEMCELL Technologies Inc., Vancouver, BC, Canada) and flushed for 5 minutes with a gas mixture of 5% CO_2_-95% N2; the final medium pO2 value was 2%. The chamber was then sealed and placed at 37°C in conventional cell incubator.

Alternately, cells were treated for 4 hours with 100µM/L of the hypoxia-mimicking CoCl_2_ (Sigma-Aldrich) which increased baseline levels of HIF-1α.

5-Azacytidine and PI3K inhibitor LY294002 were purchased from Sigma Aldrich and Cell Signaling (Danvers, MA, USA), respectively.

### In vitro transfection of MM cells with synthetic mir-199a-5p

Synthetic pre-miRNAs were purchased from Ambion (miRNA mimic MC10893, Applied Biosystems). 1×10^6^ cells were electroporated with scrambled (miR-NC) or synthetic pre-miR-199a-5p (miR-199a-5p) at a final concentration of 100nM, using Neon Transfection System (Invitrogen), with 1050 V, 30ms, 1 pulse. Cell transfection efficiency was evaluated by flow cytometric analysis of FAM™ dye-labeled synthetic miRNA inhibitor (Invitrogen) transfection.

### Quantitative real-time amplification of miRNAs and mRNAs

Total cellular RNA was extracted using TRIzol Reagent (Invitrogen, Life Technologies, Carlsbad, CA, USA) according to the manufacturer's protocol. qRT–PCR was used to confirm the expression levels of mRNAs and miRNAs.

The single-tube TaqMan miRNA (Assay ID 000498, Applied Biosystems, Life Technologies) was used to detect and quantify mature miR-199a-5p according to the manufacturer's instructions, by the use of iQ5 multicolor detection system (Bio-Rad, Berkeley, CA, USA). miR-199a-5p expression was normalized on RNU44 (Applied Biosystems, Assay Id 001094).

For mRNA detection, oligo-dT-primed cDNA was obtained using the High Capacity cDNA Reverse Transcription Kit (Applied Biosystems) and then used as template to quantify VEGF-A(hs00900054_m1), IL-8 (hs00174103_m1), IL-6(hs00985639_m1), FGFb (hs00266645_m1), MMP-2(hs01548727_m1), CXCR4(hs00237052_m1), SNAIL (hs00195591_m1), VCAM1(hs01003370_m1), ICAM1(hs00277001_m1), AKT1 (hs00920512_m1) levels by TaqMan assay (Applied Biosystems); normalization was performed with B-actin. Comparative real time polymerase chain reaction (RT–PCR) was performed in triplicate, including no-template controls. Relative expression was calculated using the comparative cross threshold (Ct) method.

### Western blotting and antibodies

SDS-PAGE and Western Blotting (WB) were performed according to standard protocols [73]. Briefly, cells were lysed in lysis buffer containing 15mM Tris/HCl pH7.5, 120mM NaCl, 25mM KCl, 1mM EDTA, 0.5% Triton X100, Halt Protease Inhibitor Single-Use cocktail (100X, Thermo Scientific). Whole lysate (50µg per lane) were separated using 4-12% Novex Bis-Tris SDS-acrylamide gels (Invitrogen), electro-transferred on Nitrocellulose membranes (Bio-Rad), and immunoblotted with the appropriate antibodies. The antibodies against the following proteins were used for the procedure: Histone H3 (D1H2), HIF-1-α (#3716), E-cadherin (clone 24E10), Phospho-Akt (Ser473), Akt (pan) (C67E7), Caspase-7 (#9492), Caspase-3 (#9662), Cleaved PARP (Asp214; D64E10) were obtained from Cell Signaling (Beverly, MA). SIRT1 from Serotec and VEGF-A (MAB293) from R&D systems; α-Tubulin (C-20), MMP2 (2C1), DDR1 (C-20), p21^Cip1^(sc-397), p27^Kip1^(sc-528), Bcl-2 (sc-7382), and all secondary antibodies were obtained from Santa Cruz Biotechnology (Santa Cruz Biotechnology, Inc., Santa Cruz, CA, USA). Chemiluminescence was detected using Pierce ECL Western Blotting Substrate (cat. 32109, Pierce, USA).

### Nuclear fraction-enrichment

Cells were lysed in cytoplasm lysis buffer A (10mM HEPES pH 7.9, 10mM KCl, 0.2mM EDTA and 1mM DTT), containing protease inhibitors, 0.5mM phenylmethylsulfonylfluoride (PMSF) and 0.6% Nonidet P-40 (Sigma Chemical Co., St. Louis, MO, USA). Lysates were centrifuged at 10,000 r.p.m. for 10 min at 4°C, and the supernatants (cytoplasmic fractions) were split into aliquots and rapidly frozen. The nuclear pellet was washed in buffer A without Nonidet P-40 and finally resuspended in nuclear lysis buffer B (20mM HEPES pH 7.9, 0.4M NaCl, 2mM EDTA, 1mM DTT), containing protease inhibitors and 1mM PMSF (Sigma Chemical Co.). Samples were incubated on ice for 30 min and centrifuged at 13 000 r.p.m. for 10 min at 4 °C; the supernatants (nuclear fractions) were split into aliquots and rapidly frozen. The remaining pellets, containing DNA as well as proteins tightly associated with DNA, were washed in reticulocyte buffered buffer (RSB: 10mM NaCl, 10mM Tris-HCl pH 7.5 and 1.5mM MgCl2) and finally resuspended in RSB and HCl 0.4N to obtain HCl-soluble proteins [74, 75]. The samples were incubated at least 1 h at 4 °C and centrifuged at 10 000 r.p.m. for 20 min at 4 °C. The acid-soluble proteins were recovered from the supernatants by precipitation with 10 volumes of acetone at -20°C and centrifugation at 10,000 r.p.m. for 20 min at 4 °C. HCl-soluble proteins were finally resuspended in distilled water, split into aliquots and frozen.

### Cell proliferation

For cell proliferation analysis, MM cells were plated in 6 well plates, electroporated with miR-199a-5p or miR-NC and then harvested, plated at 1,5-2,0×10^5^/ml, and counted at 24 hours intervals using a Trypan Blue-exclusion assay.

### Annexin v apoptosis assays

Annexin V apoptosis assays were performed using Annexin V-FITC kit (Biolegend, San Diego, CA, USA) according to manufacturer's protocol. The stained cells were immediately analyzed on a FACScalibur flow cytometer (Becton Dickinson). The data were presented as the percentage of apoptotic cells.

### Chemotaxis assay

Chemotaxis was determined using 8-u-pore filters for the Transwell migration assay (BD Biosciences), according to the manufacturer's instructions.

Briefly, OPM2 cells (1×10^5^/well) transfected with either scrambled (miR-NC) or synthetic pre-miR-199a-5p (miR-199a-5p) were washed and resuspended in RPMI 1640 medium supplemented with 1% FBS. Then cells were placed in the upper migration chambers, whereas the lower chamber contained RPMI 1640 medium supplemented with 10% FBS. After 5 hours of incubation at 37°C-5%CO_2_, cells migrated to the lower chambers were determined by a Trypan-Blue exclusion assay. Huvec (7×10^3^ cells/well) were resuspended in serum free RPMI 1640 medium supplemented with 0.1% BSA in transwell chemotaxis (BD Biosciences) above 8 um pore filters and exposed to conditioned medium from multiple myeloma cells transfected with either scrambled (miR-NC) or synthetic pre-miR-199a-5p (miR-199a-5p).

Filters were removed after 6 hours, fixed in methanol and stained with Diff-Quick (Medion Diagnostics GmbH). Two independent experiments were carried out in triplicate; cells from five different fields were counted for each condition.

### Adhesion assay

HS-5 stromal cells were grown to confluence in 12-well plates and fixed with glutaraldehyde 0.0125% (Agar Scientific LTD). After fixation, cells were treated with 10 mM ethanolamine to block aldehydic groups and washed several times before plating MM cells.OPM2 cells (0.5×10^6^ cells/well) transfected with either scrambled (miR-NC) or synthetic pre-miR-199a-5p (miR-199a-5p) were added to each well and incubated for 4 hours at 37°C-5%CO_2_. Non adherent cells were washed and adhesion was detected by hematoxylin/eosin staining. Each test group was assayed in triplicate; five fields were counted for each condition.For cells cultured under hypoxic conditions, all procedures were performed in the hypoxic chamber (STEMCELL Technologies Inc.).

### Luciferase reporter experiments

The 3'UTR of HIF-1α were cloned in pEZX-MT01 vector and purchased from Genecopoeia. MM cells were electroporated as above described using 10µg of the firefly luciferase reporter; for each plate, 100 nM of the synthetic miR-199a-5p or miR-NC were used. Firefly and Renilla luciferase activities were measured consecutively using the dual-luciferase assay kit (Promega Corporation, Madison, WI, USA) 24 hours after transfection.

Data are expressed as luminescence from firefly luciferase divided by luminescence from renilla luciferase.

### Animals and in vivo models of human MM

Male CB-17 non-obese diabetic/severe combined immunodeficient (NOD.SCID) mice (6- to 8-weeks old; Harlan Laboratories, Inc., Indianapolis, IN, US) were housed and monitored in our Animal Research Facility. All experimental procedures and protocols had been approved by the Institutional Ethical Committee and conducted according to protocols approved by the National Directorate of Veterinary Services (Italy). In accordance with institutional guidelines, mice were sacrificed when their tumors reached 2 cm in diameter or in the event of paralysis or major compromise in their quality of life, to prevent unnecessary suffering. To obtain xenograft model of human MM, 5×10^6^ NCI-H929 cells in 100 µL RPMI-1640 medium were subcutaneously injected into the interscapular area of NOD-SCID mice. Treatments were initiated after the detection of palpable tumors, (approximately 30 mm^3^), about 10 days following the injection of MM cells. The tumor sizes were measured as previously described [30] Mice were randomized into 2 groups and treated with synthetic miR-199a-5p mimics (Life Technologies) or scramble control (miR-NC). Each dose contained 20 µg synthetic oligo, which equals 1mg/kg per mouse with an average weight of 20 g. Administration of miRNAs mimics was performed by the use of the neutral lipid emulsion (NLE) (MaxSuppressor *in vivo* RNA Lancer II, BIOO Scientific, Austin, TX) as previously described [76]. Treatments were performed intratumorally (i.t.) every three days for a total of six injections.

### Elisa

VEGF-A levels secreted by MM cells were quantified by VEGF–enzyme-linked immunosorbent assays (Quantikine ELISA) (R&D Systems, Minneapolis, MN), according to the manufacturer's recommendations.

Briefly, OPM2 cells (1×10^6^ cells/well) transfected with either scrambled (miR-NC) or synthetic pre-miR-199a-5p (miR-199a-5p) were added to each well in RPMI supplemented with 1% FBS and cultured in both normoxic and hypoxic conditions. Supernatants were harvested after 48 hours and analyzed.

### Statistical analysis

The data were expressed as means±s.d. Differences were assessed by two tailed Student's t-test using the Excel software. P value < 0.05 was considered to be statistically significant.

## SUPPLEMENTARY FIGURES AND TABLE




