# Supplementary material for: Targeting of multiple myeloma-related angiogenesis by miR-199a-5p mimics: in vitro and in vivo anti-tumor activity
Source: Oncotarget. 2014 Mar 14;5(10):3039–54. doi: 10.18632/oncotarget.1747 (PMC4102790; doi:10.18632/oncotarget.1747)
Supplement: Supplementary file 1 [file oncotarget-05-3039-s001.pdf]

Targeting of multiple myeloma-related angiogenesis by miR-199a-5p mimics: in vitro and in vivo anti-tumor activity – Raimondi et al

A

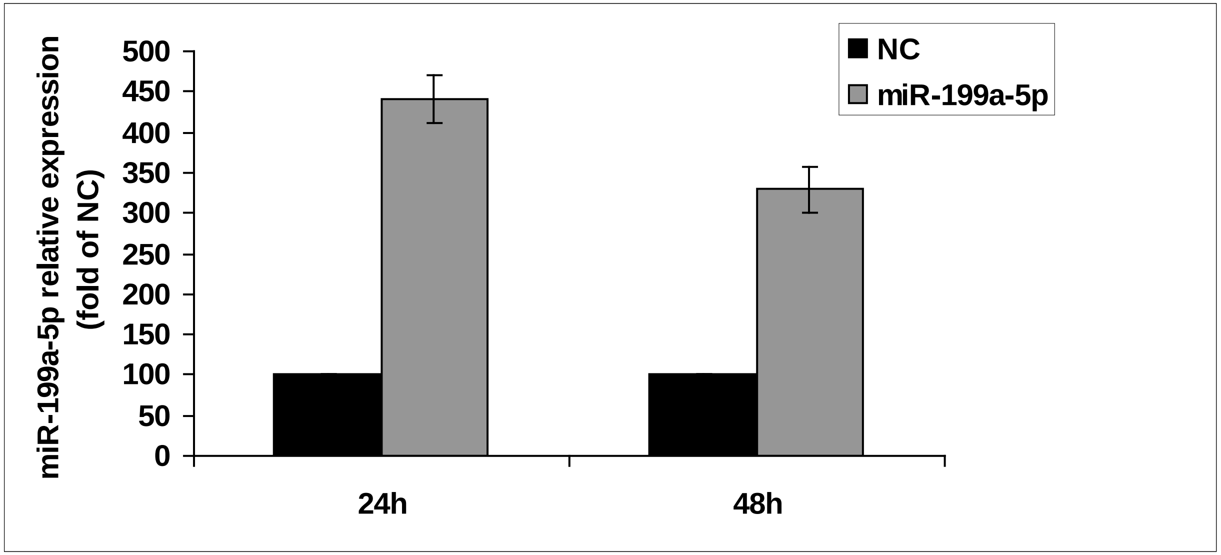

Supplemental Figure 1: Level of miR-199a-5p after transfection in MM cells. (**a**) Level of miR-199a-5p 24 and 48 hours after transfection with synthetic miR-199a-5p (miR-199a-5p) or scrambled oligonucleotides (NC) in OPM2 cell line. Raw Ct were normalized to RNU44 housekeeping snoRNA and expressed as fold increase over negative control (black column, 1 arbitrary unit). Columns, means; Bars, S.D. Values represent mean of three different experiments.

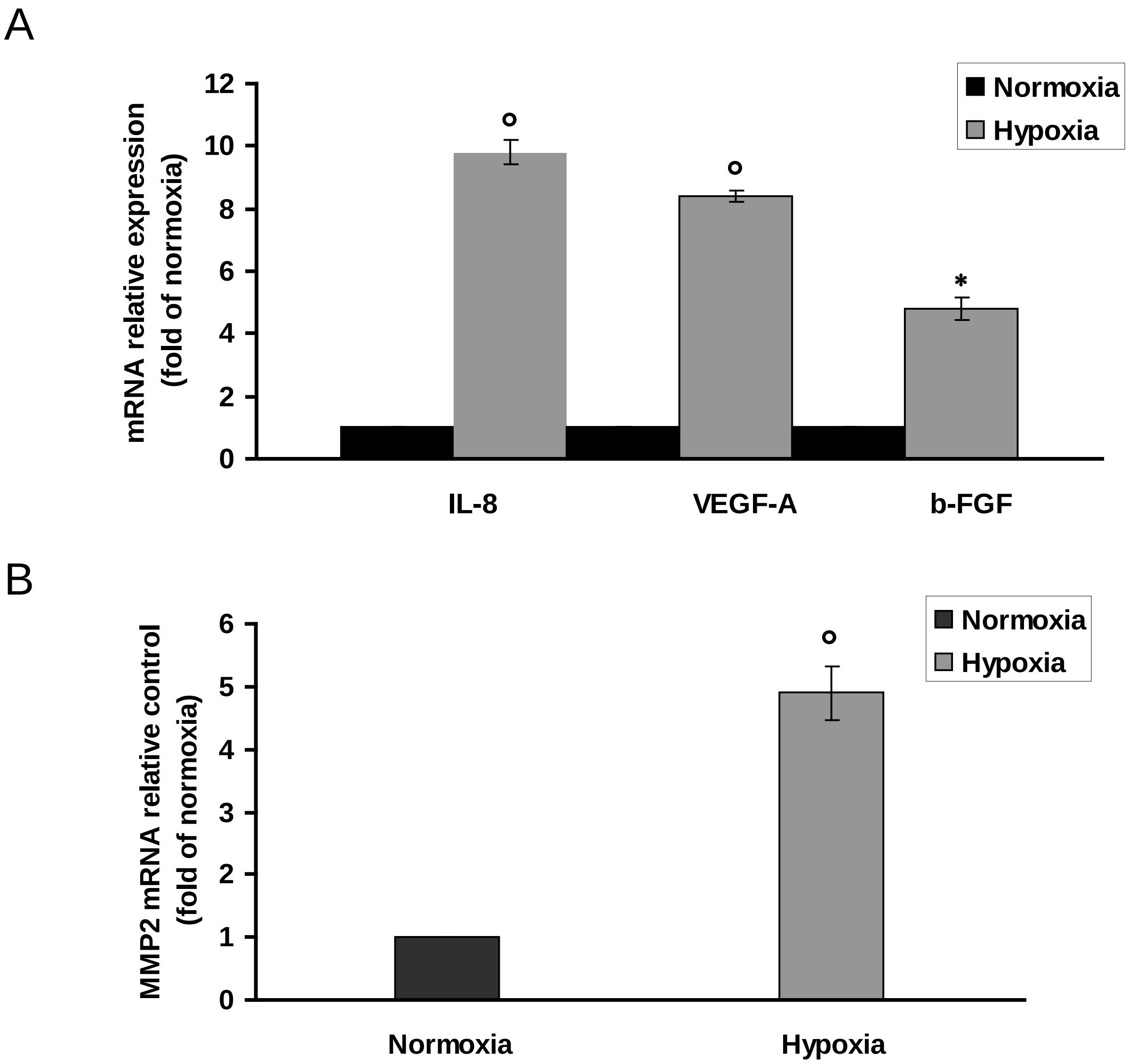

Supplemental Figure 2: Hypoxia increases the expression of pro-angiogenic and matrix metalloproteinase genes in MM cells. (**a**) Quantitative RT-PCR of VEGF-A, IL-8 and b-FGF in both normoxic and hypoxic OPM2 cells. Raw Ct were normalized to b-actin housekeeping and expressed as fold increase of normoxia (black column, 1 arbitrary unit). Columns, means; Bars, S.D. Values represent mean of three different experiments. °P<0,01; ∗ P<0,05. (**b**) Quantitative RT-PCR of matrix metalloproteinase-2 (MMP2) in both normoxic and hypoxic OPM2 cells. Raw Ct were normalized to b-actin housekeeping and expressed as fold increase of normoxia (black column, 1 arbitrary unit). Columns, means; Bars, S.D. Values represent mean of three different experiments. °P<0,01.

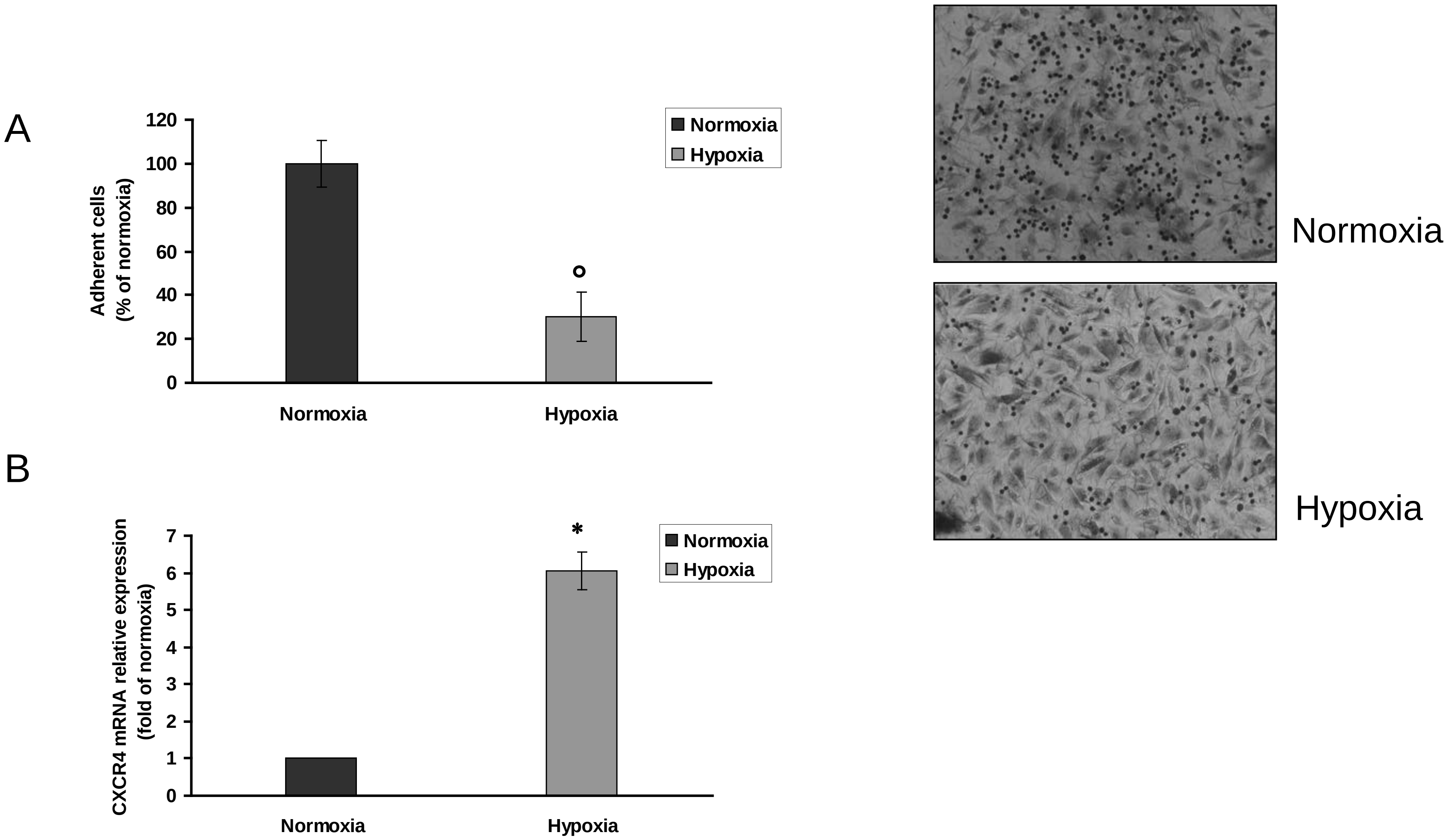

Supplemental Figure 3: Hypoxia decreases adhesion of MM cells to a monolayer of BMSCs under hypoxic conditions and influences CXCR4 expression (**a**) Adhesion assay of both normoxic and hypoxic OPM2 seeded on to hypoxic BMSCs monolayer (right panel); OPM2 cells treated as in (a) and observed at contrast phase microscopy (left panel). °P<0,01. (**b**) Quantitative RT-PCR of CXCR4 in both normoxic and hypoxic OPM2 cells. Raw Ct were normalized to b-actin housekeeping and expressed as fold increase of normoxia (black column, 1 arbitrary unit). Columns, means; Bars, S.D. Values represent mean of three different experiments. * P<0,05.

A

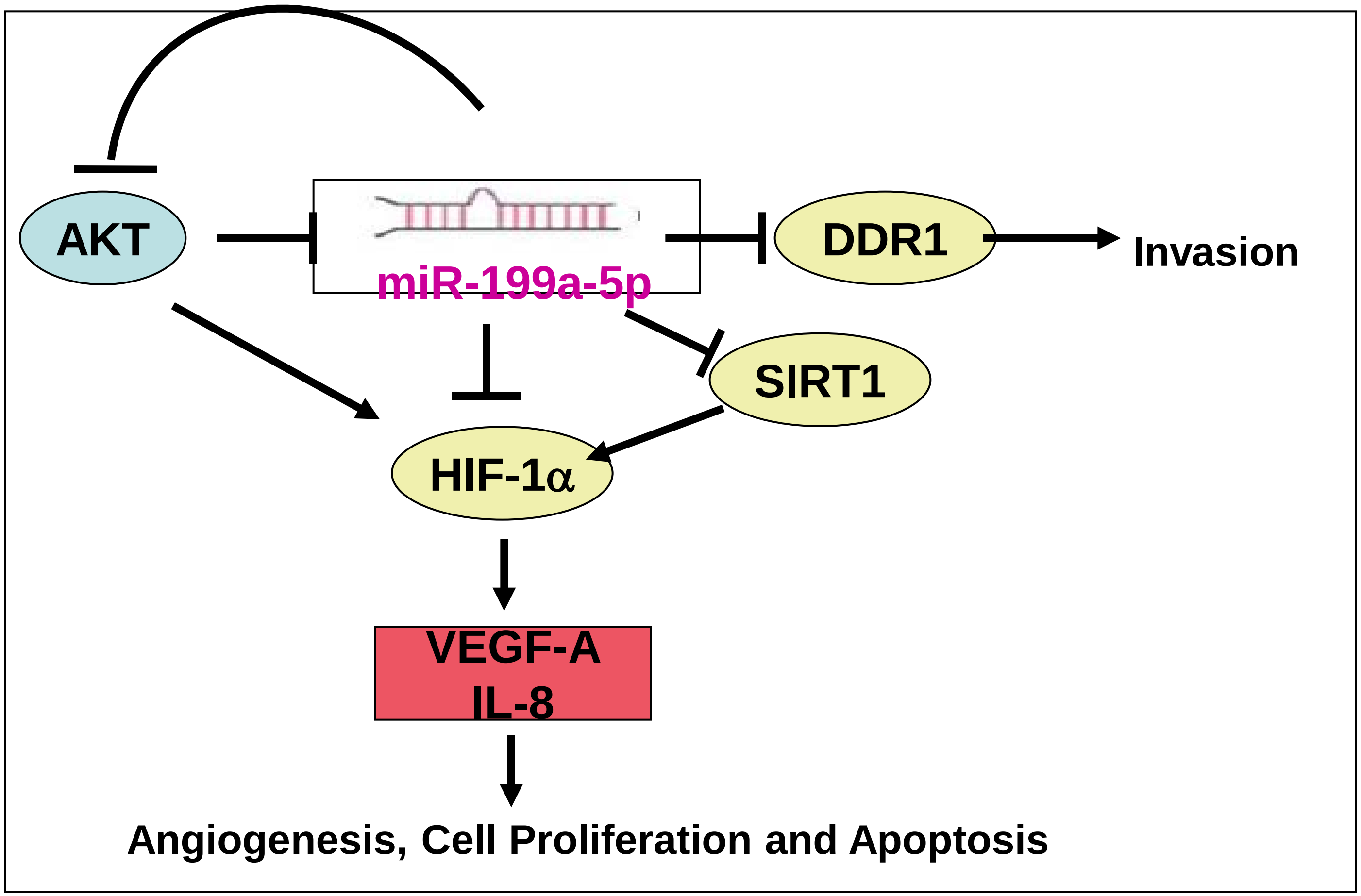

Supplemental Figure 4: AKT\miR-199a-5p\HIF-1a loop in MM (**a**) Cartoon showing the interplay between miR-199a-5p\HIF1a and AKT in a regulatory feedback loop.

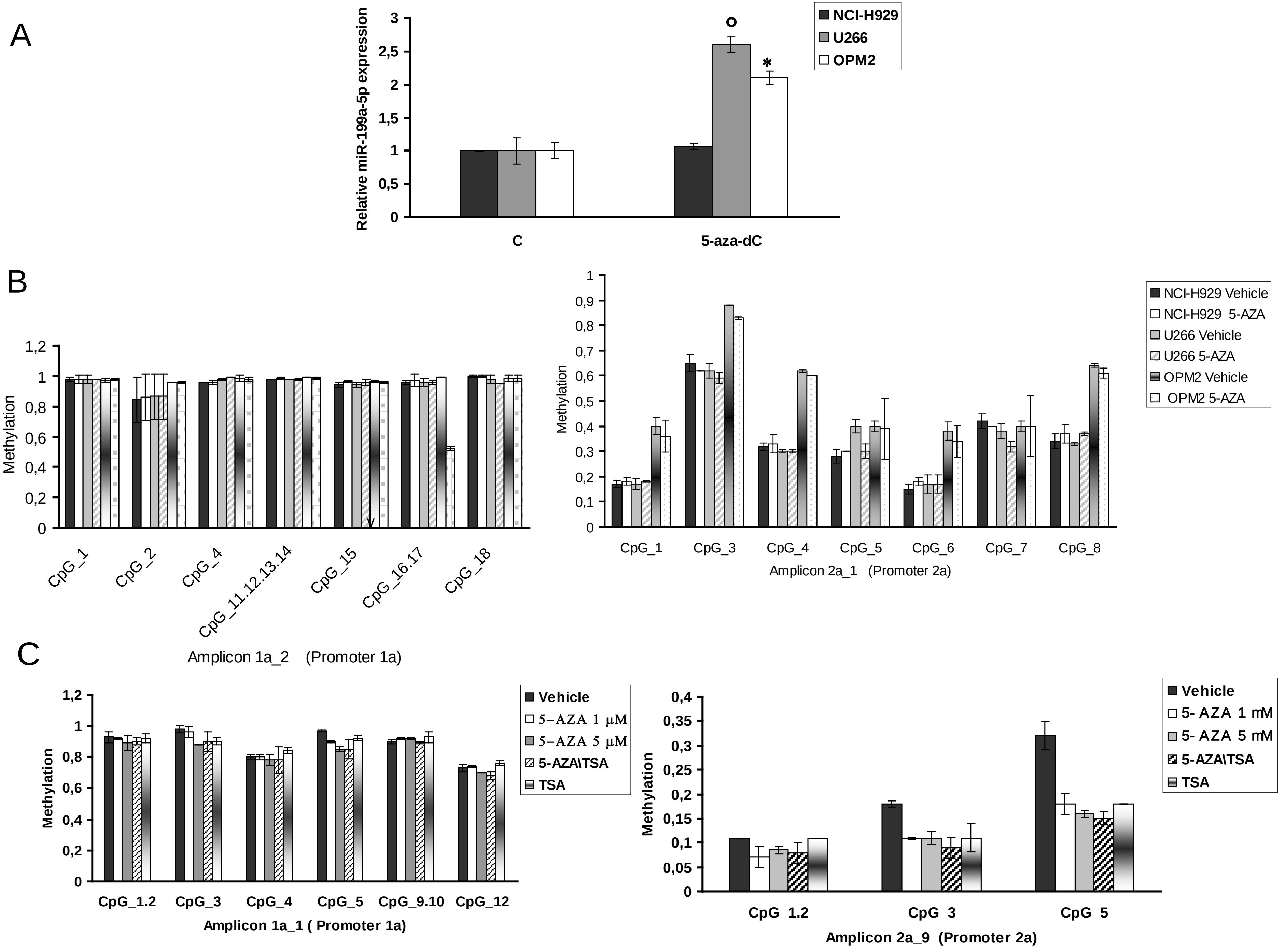
A
Relative miR-199a-5p expression
3
2,5
2
1,5
1
0,5
0
C
5-aza-dC
NCI-H929
U266
OPM2
B
Methylation
1,2
1
0,8
0,6
0,4
0,2
0
CpG_1
CpG_2
CpG_4
CpG_11.12.13.14
CpG_15
CpG_16.17
CpG_18
Amplicon 1a_2 (Promoter 1a)
Methylation
1
0,9
0,8
0,7
0,6
0,5
0,4
0,3
0,2
0,1
0
CpG_1
CpG_3
CpG_4
CpG_5
CpG_6
CpG_7
CpG_8
Amplicon 2a_1 (Promoter 2a)
NCI-H929 Vehicle
NCI-H929 5-AZA
U266 Vehicle
U266 5-AZA
OPM2 Vehicle
OPM2 5-AZA
C
Methylation
1,2
1
0,8
0,6
0,4
0,2
0
CpG_1.2
CpG_3
CpG_4
CpG_5
CpG_9.10
CpG_12
Amplicon 1a_1 (Promoter 1a)
Vehicle
5-AZA 1 μM
5-AZA 5 μM
5-AZA\TSA
TSA
Methylation
0,4
0,35
0,3
0,25
0,2
0,15
0,1
0,05
0
CpG_1.2
CpG_3
CpG_5
Amplicon 2a_9 (Promoter 2a)
Vehicle
5- AZA 1 mM
5- AZA 5 mM
5-AZA\TSA
TSA

Supplemental Figure 5: Effects of demethylating agents on methylation levels of the two promoter regions in chromosome 19 (1a) and in chromosome 1 (2a) of miR-199a-5p. (a) Quantitative RT-PCR of miR-199a-5p in NCI-H929, OPM2 and U266 MM cell lines after 72 hours of 1mM 5-aza-dC treatment. Raw Ct were normalized to RNU44 housekeeping snoRNA and expressed as fold increase over untreated cells (black column, 1 arbitrary unit). Columns, means; Bars, S.D. Values represent mean of three different experiments. °$P<0,01$. ∗ $P<0,05$. **(b)** Mean methylation levels of CpG sites located in the two promoter regions in chromosome 19 (1a) and in chromosome 1 (2a) of miR-199a-5p, as determined by Sequenom MassARRAY analysis, in NCI-H929, U266 and OPM2 cells treated for 72 hours with vehicle or 1mM 5-azacytidine. Data are expressed as mean ± SD of two independent experiments performed in triplicate. Two amplicons representative are shown. **(c)** Mean methylation levels of CpG sites located in the two promoter regions in chromosome 19 (1a) and in chromosome 1 (2a) of miR-199a-5p, as determined by Sequenom MassARRAY analysis, in U266 cells treated for 72 hours with either 5μM azacytidine or 100 ng\ml trichostatin A (TSA). Data are expressed as mean ± SD of two independent experiments performed in triplicate. Two amplicons representative are shown.
