# Supplementary material for: Targeting of multiple myeloma-related angiogenesis by miR-199a-5p mimics: in vitro and in vivo anti-tumor activity
Source: Oncotarget. 2014 Mar 14;5(10):3039–54. doi: 10.18632/oncotarget.1747 (PMC4102790; doi:10.18632/oncotarget.1747)
Supplement: Supplementary file 2 [file oncotarget-05-3039-s002.pdf]

Targeting of multiple myeloma-related angiogenesis by miR-199a-5p mimics: in vitro and in vivo anti-tumor activity – Raimondi et al

	**Amplicon**	**Primer Sequence**	**Annealing Temperature (°C)**
Promoter 1a	Amplicon 1	For 5'- **AGGAAGAGAG**AATAGGTAGTTTGAATATTGGGTTGG -3'<br>Rev 5'- **CAGTAATACGACTCACTATAGGGAGAAGGCT**AAAAATCAAACATTCCTCCTAAACC -3'	58
	Amplicon 2	For 5'- **AGGAAGAGAG**TATTTGGAGAAAGTTTTGGGTTTTT -3'<br>Rev 5'- **CAGTAATACGACTCACTATAGGGAGAAGGCT**CAAACCATTCCAACTAATACCACAT -3'	52
	Amplicon 3	For 5'- AGGAAGAGAGATGTGGTATTAGTTGGAATGGTTTG -3'<br>Rev 5'- **CAGTAATACGACTCACTATAGGGAGAAGGCT**AATAACACAAAACCTAAATCCCTCC -3'	58
Promoter 2a	Amplicon 2	For 5'- **AGGAAGAGAG**TGGGGTTTATTTTTTGTTTAGTTGA -3'<br>Rev 5'- **CAGTAATACGACTCACTATAGGGAGAAGGCT**ACAAAATCTCCAAAAACTTCCTTCT -3'	52
	Amplicon 9	For 5'- **AGGAAGAGAG**AGAAGGAAGTTTTTGGAGATTTTGT -3'<br>Rev 5'- **CAGTAATACGACTCACTATAGGGAGAAGGCT**ATCTTCTCCTTAAAAACAACCCATT -3'	52

T7-promoter tag and 10-mer tag sequence added to the reverse and forward primers, respectively, are indicated in bold.
